# Hydrophobically Modified Polyacrylamide Incorporating Both Hydrophilic and Hydrophobic Units: Enhanced Printability and Stability in Aqueous Ink

**DOI:** 10.3390/molecules29215105

**Published:** 2024-10-29

**Authors:** Zhi-Rui Liu, Li-Lin Tan, Juan Gao, Zi-Ye Qin, Xin-Xin Huo, Zhi-Min Liang

**Affiliations:** 1 Shantou Engineering Technology Research Center for Green and Precise Manufacturing of High-Value Chemicals, Chemistry and Chemical Engineering Guangdong Laboratory, Shantou 515031, China; liuzr@ccelab.com.cn (Z.-R.L.); 22jgao@stu.edu.cn (J.G.); liangzm@ccelab.com.cn (Z.-M.L.); 2 College of Chemistry and Chemical Engineering, Shantou University, Shantou 515063, China

**Keywords:** hydrophobic association, polyacrylamide, aqueous ink, printability, stability

## Abstract

For this research, three hydrophobically modified polyacrylamides, HPAAB, HPAAF, and HPAAS, with multiple hydrophobic monomers were designed, synthesized, and used as thickeners in aqueous ink for digital ink-jet printing. The structures were characterized by Fourier transform infrared (FTIR) analysis and nuclear magnetic resonance (NMR) spectroscopy. The viscosity–average molecular weight was determined by intrinsic viscosity determination and was adjusted according to hydrophobic content. The critical association concentration (CAC) of polymers was measured simultaneously using the apparent viscosity method and the fluorescence spectrum. The formation of a network structure and the mechanism of hydrophobic association are visualized dynamically with a scanning electron microscope (SEM) at different concentrations. Under the same conditions, HPAAB exhibited excellent thickening ability across different pH levels, temperatures, and shear rates, which is caused by the longer hydrophobic side chain and the stronger hydrophobic effect of the behenyl polyoxyethylene ether methacrylate (BEM) group. Furthermore, an aqueous ink using HPAAB as a thickener displays significant printability and stability, functioning much better than a corresponding aqueous ink that uses a commercial thickener. This is the first example of a hydrophobic associating polyacrylamide, incorporating both hydrophilic and hydrophobic units within a single hydrophobic chain, thereby serving as an efficient thickener for aqueous ink.

## 1. Introduction

Digital ink-jet printing is a technology based on sending digitized pattern data to an ink-jet printer, then jetting ink droplets to directly print the digitized pattern onto a substrate [1]. Compared with traditional graphic printing technologies, this method offers the advantages of small-batch production, significant variety, multifunctionality, and personalized customization [2]. However, most of the inks used in digital ink-jet printing are based on organic solvents and contain volatile organic compounds (VOCs) to maintain dispersion stability and enhance printability. Organic solvent-based ink can cause environmental pollution and may pose a risk to human health during the production process. In contrast, using water as a solvent in ink can reduce VOCs emissions and leave minimal solvent residue [3]. Therefore, there is a need to develop environmentally friendly and functional aqueous inks as a substitute for organic inks in ink-jet printing.

In the ink-jet printing process, it is necessary to use a thickener and dye solution to form a color paste that suppresses the diffusion of ink droplets once on the substrate, resulting in more accurate and stable printed designs. For example, in textile printing, a thickener is used as a carrier to transfer dye to the fabric [4]. This improves the dispersion and adhesion of dye particles, regulates ink flow characteristics and enhances printing accuracy [5]. The rheological and thixotropic properties of ink influence the clarity of printed pattern contours, while the particle size and dispersion of dyes can affect the continuity of ink-jet printing processes. A small amount of undissolved pigment particles in the ink may cause nozzle clogging. The dispersed particles are prone to agglomeration or even precipitation at the bottom of the ink container, due to the low viscosity of the ink. Among all the components of water-based ink, thickeners play a crucial role in thickening, dispersing, and stabilizing, which is closely related to process performance and storage stability.

The current printing thickeners used in printing mainly include natural thickeners and their derivatives [6,7,8], synthetic thickeners, and composite thickeners [9,10]. Natural thickeners have significant limitations regarding source, purity, and stability. For example, cellulose natural thickeners exhibit poor thickening performance and are prone to biodegradation. Synthetic thickeners mainly include polyacrylic acids [11,12], polyurethanes [13,14], and others. Polyurethane has poor thickening performance due to its structural properties. The molecular chain of polyacrylate thickener contains a large number of carboxyl groups, which can stretch the polymer chains and increase the viscosity of the solution under alkaline conditions. In addition, these carboxyl groups make the viscosity of the solution sensitive to acids, alkalis, and electrolytes. To further enhance the thickening performance of polymers, novel hydrophobic-associating thickeners were developed by introducing less than 2 mol% of hydrophobic groups into a hydrophilic backbone [15]. These mainly include hydrophobically modified polyurethane (HEUR), alkali swellable emulsion (HASE), and polyacrylamide. The unique rheological properties and hydrophobic moieties of hydrophobically modified polymers make them highly effective in a range of applications, including petroleum [16,17,18], coatings [19,20], sewage treatment [21,22], biomedical [23,24], and other fields. Both HEUR and HASE have been commercialized as components of water-based inks [13,14,25,26,27]. HEUR is a non-ionic rheological modifier with a high production cost, due to its complex synthesis process. In contrast, HASE offers good thickening performance at a lower cost; however, its thickening efficiency is highly dependent on pH, latex composition, and ink solid content. The development of high-performance, low-cost thickeners remains a key issue in the current ink industry.

Hydrophobically associating polyacrylamide (HAPAM), another important synthetic thickener, has been extensively studied over the past few decades due to its unique characteristics, which include water solubility and high levels of thickening activity [28,29,30,31]. The hydrophobic microdomains in HAPAM enhance the formation of physical cross-linked network structures between polymer chains. Therefore, HAPAM has been employed as an efficient thickener in many fields, including fracturing, drilling, latex paint systems, etc. [32,33,34,35,36,37,38]. However, such thickeners are rarely reported in ink research, especially regarding digital ink-jet printing. The research on HAPAM polymers in the literature mainly focuses on high molecular weight (ranging from millions to tens of millions) and completely hydrophobic monomers, which are unsuitable for use in ink-jet printing [33]. The high polymer molecular weight increases the entanglement of molecular chains, thereby enhancing their thickening capabilities in solutions. However, this also makes them unsuitable for low-viscosity and high-fluidity solution systems. Therefore, maintaining good thickening ability at an appropriate molecular weight remains a considerable challenge.

Herein, inspired by this concept, we developed moderate molecular-weight hydrophobically modified polyacrylamide as a thickener, incorporating both hydrophilic and hydrophobic fragments within the hydrophobic chain. The thickening performance of HAPAM can be adjusted by changing the size of the hydrophobic chain and the degree of hydrophobic associations. In this article, the viscosity-average molecular weight, CAC, and the effect of pH, temperature, and shear rate on viscosity are systematically investigated. The thickening mechanism for hydrophobic associating polymers is explored using SEM techniques. Given that the characteristics of ink depend on various factors, including latex composition, particle size, the stabilization system used, and pigment, we have developed an ink formulation system using highly dispersed commercial colorants. The ink is thoroughly dispersed before testing to minimize the impact of pigment agglomeration on its properties. The printability and stability of ink formulated with HAPAM thickener are studied by comparing it with commercial thickeners. This study explores the potential of using HAPAM with strong hydrophobically associating properties in the development of water-based inks.

## 2. Results and Discussion

### 2.1. Synthesis and Structural Characterization of HAPAM

**Figure 1:**
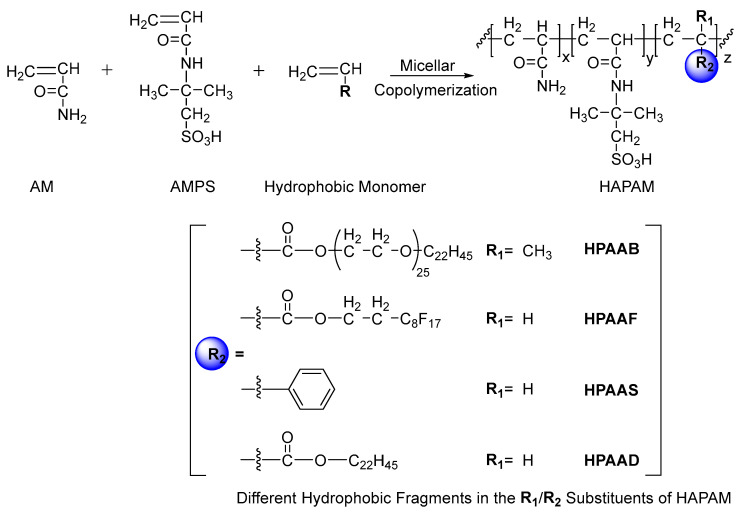
Synthesis of polyacrylamide copolymers with different hydrophobic units.

The general synthetic composition of HAPAM is outlined in Figure 1. For these experiments, HAPAM was synthesized with different hydrophobic monomers such as BEM, perfluorooctyl ethyl acrylate (FEA), and styrene (ST) through micellar copolymerization, with the mixtures named HPAAB, HPAAF, and HPAAS, respectively. The perfluoroalkyl chain in HPAAF and the phenyl ring in HPAAS are completely hydrophobic, whereas an HPAAB featuring alkyl and the polyethylene glycol chain in the BEM chain exhibits both hydrophilic and hydrophobic characteristics. Acrylamide (AM) and 2-acrylamido-2-methyl-1-propanesulfonic acid (AMPS) were used as the common monomers in all HAPAMs. The anionic sulfonic group in AMPS offers good solubility and salt resistance, due to its strong polarity; in addition, sulfonate anions generate electrostatic repulsion in aqueous solutions, which results in more stretched polymer molecular chains [39].

The HAPAM copolymers were prepared by free radical polymerization in a one-pot reaction, using sodium dodecyl sulfonate (SDS) as the surfactant and potassium persulfate as the initiator. Prior to polymerization, the AM, AMPS, and surfactant were added and dissolved in deionized water while mixing to achieve a uniform micellar solution. Following this stage, the hydrophobic monomers were added individually and stirred continuously until a transparent, uniform mixture was achieved, which is key for micellar copolymerization. Finally, after the addition of an initiator, the generation of free radicals triggered the reaction between the hydrophilic and hydrophobic micelles at the interface, leading to the formation of polymers. It is important to control the solid content of the solution during synthesis as the increasing polymer molecular weight raises the viscosity, making it difficult to stir the reaction mixture. The detailed differential scanning calorimetry (DSC) analysis confirmed the presence of hydrophobic microblock structures in the polymer chain (Figure S1), which is one of the main factors used to determine the hydrophobic association interactions of HAPAM [40]. Details of the procedure of HAPAM synthesis are given in the Supplementary Information.

**Figure 2:**
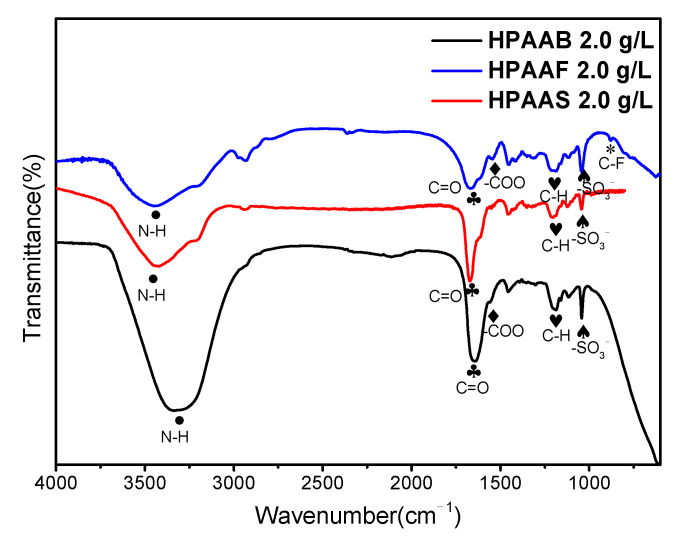
The FTIR spectra of HPAAB, HPAAF, and HPAAS.

The structure of HPAAB, HPAAF, and HPAAS was confirmed using FTIR analysis and NMR spectroscopy techniques. The absorption peaks at 3325.31, 3434.86, and 3440.98 cm^−1^, as shown in Figure 2, represent stretching vibrations due to N-H. Along with the absorption peaks at 1644.07, 1671.14, and 1667.48 cm^−1^ in the three curves assigned to the C=O stretching vibrations, the absorption peaks at 1039.51, 1039.56, and 1040.86 cm^−1^ represent the symmetrical stretching vibrations of –SO_3_^−^. The absorption peak at 879.85 cm^−1^ represents the stretching vibrations of the C-F group in the HPAAF polymer, while the absorption peaks at 1554.28 and 1540.09 cm^−1^ are the stretching vibrations of the -COO group in the HPAAB and HPAAF samples [41]. The ^13^C-NMR signals at 17.92, 34.70, 26.35, 179.54, and 41.94 ppm are assigned to the –CH_3_, –CH_2_–, –CH–, C=O, C–N, and C–S in HPAAB (Figure S2). Similar characteristic peaks are used to analyze the structures of HPAAF (Figure S3) and HPAAS (Figure S4). The signals at 57.53 ppm and 58.84 ppm correspond to the C–O in HPAAB and HPAAS, while the signal at 127.85 ppm is assigned to the benzene ring in HPAAS. The ^19^F-NMR signals at 79.33 and 122.25 ppm can be attributed to the –CF_3_ and –CF_2_– in HPAAF, respectively. The results of FTIR and NMR confirm that HPAAB, HPAAF, and HPAAS were the target products. Detailed structural assignment information is listed in the Supplementary Information.

### 2.2. Viscosity-Average Molecular Weight of HAPAM

**Figure 3:**
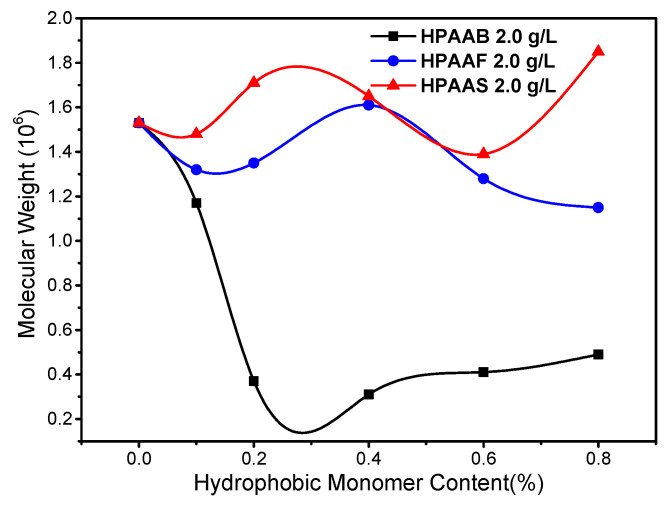
The viscosity-average molecular weight of HPAAB, HPAAF, and HPAAS at different hydrophobic monomer molar ratios.

Figure 3 shows variations in the viscosity-average molecular weight of HAPAM with different hydrophobic monomer contents. The viscosity-average molecular weight was measured by the viscosity method, using the Ubbelohde viscometer. When the hydrophobic monomers were added to the HAPAM polymers, the molecular weight gradually decreased. Due to the long branch chain and large steric hindrance effect of the BEM monomer, the molecular weight of HPAAB polymer is much lower than that of HPAAF and HPAAS at the same hydrophobic monomer molar ratios. The maximum molecular weights of HPAAB, HPAAF, and HPAAS are achieved at hydrophobic monomer molar ratios of 0.3–0.4%. In contrast, the apparent viscosity of these three polymers peaks at a 0.4% molar ratio of hydrophobic monomers (Figure S5). Therefore, the hydrophobic monomers were maintained at a molar ratio of 0.4% (*n*/*n*) in the subsequent measurements, corresponding to mass ratios of 5.76%, 2.07%, and 0.42% (*w*/*w*) in HPAAB, HPAAF, and HPAAS, respectively.

### 2.3. Properties of HAPAM

#### 2.3.1. Measurements of CAC

**Figure 4:**
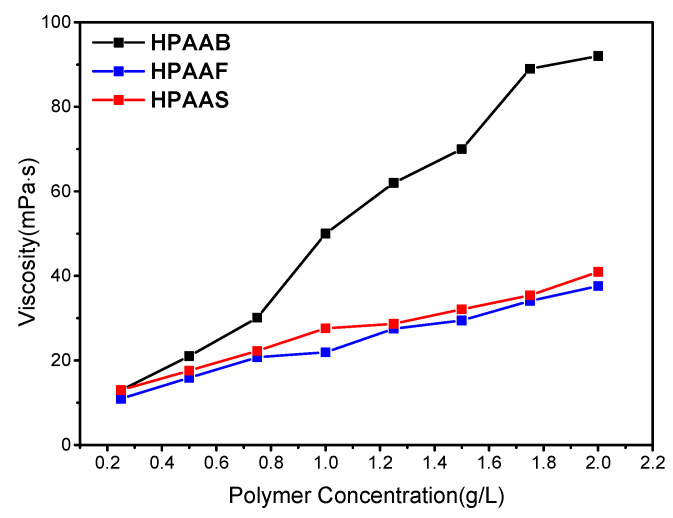
The relationship of concentration with the apparent viscosity of HPAAB, HPAAF, and HPAAS.

When the polymer concentration exceeds the CAC, a supramolecular structure that is mainly composed of intermolecular associations forms in the solution, resulting in a significant increase in fluid dynamics volume and, as a result, increased apparent viscosity. A series of polymer solutions were prepared with varying concentrations and their viscosities were recorded using a viscometer at a specific rotational speed (Figure 4). The CAC values of HPAAB, HPAAF, and HPAAS were determined to be 0.8 g/L, 1.0 g/L, and 1.3 g/L, respectively, as indicated by the abrupt increase in the apparent viscosity-concentration curve. For the same molar ratio of hydrophobes in the polymer, the lower CAC of a polymer indicates stronger intermolecular hydrophobic interactions in the solution. When the concentration exceeds the CAC value, intermolecular agglomeration dominates over intramolecular agglomeration. A three-dimensional network structure forms, leading to a significant enhancement of the apparent viscosity.

**Figure 5:**
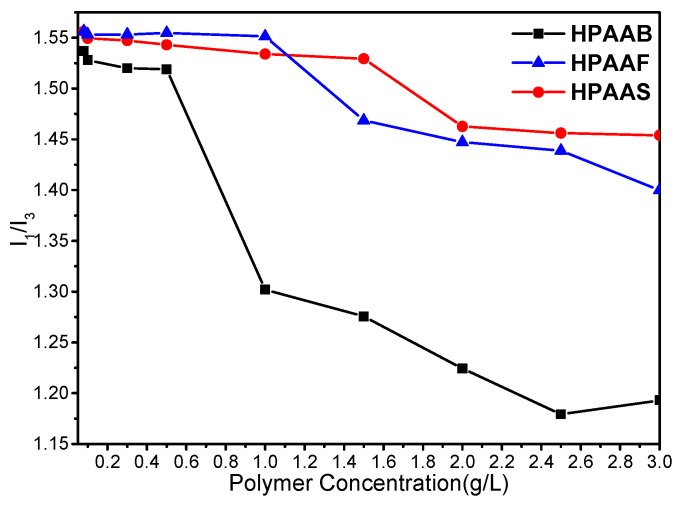
The influence of solution concentration on the I_1_/I_3_ of HPAAB, HPAAF, and HPAAS.

The change in the apparent viscosity of HAPAM solutions at different concentrations reflects the hydrophobic association behavior of HAPAM macroscopically, while the fluorometry results provide insight into this behavior at the molecular level. The effect of solution concentration on the fluorescence intensity of polymers is shown in Figure 5. The intensity ratio of the I peak (I_1_) λ at 372 nm to the III peak (I_3_) λ at 383 nm in the fluorescence spectrum is strongly dependent on the polarity around the pyrene probe [42]. Due to its low solubility in water, pyrene easily migrates from the water to the hydrophobic microdomains formed by association [43]. A weak polarity in the microenvironment around the pyrene molecule results in a lower value of I_1_/I_3_ [44]. As shown in Figure 5, the values of I_1_/I_3_ increase gradually in the range of 200–500 mg/L and remain constant at around 500 mg/L, indicating that small amounts of intramolecular hydrophobic microdomains form in HAPAM aqueous solution at low concentrations. As the concentrations of HPAAB, HPAAF, and HPAAS increase, high hydrophobic microdomains develop [45], leading to a drastic decrease in I_1_/I_3_ values in the ranges of 0.6–1.0 g/L, 0.8–1.2 g/L, and 1.2–1.5 g/L. This indicates a weakening of the polarity in the microenvironment surrounding the pyrene molecules. The fluorescence spectrum is highly consistent with the results of Figure 4.

#### 2.3.2. Hydrophobic Associating Mechanism of HAPAM

**Figure 6:**
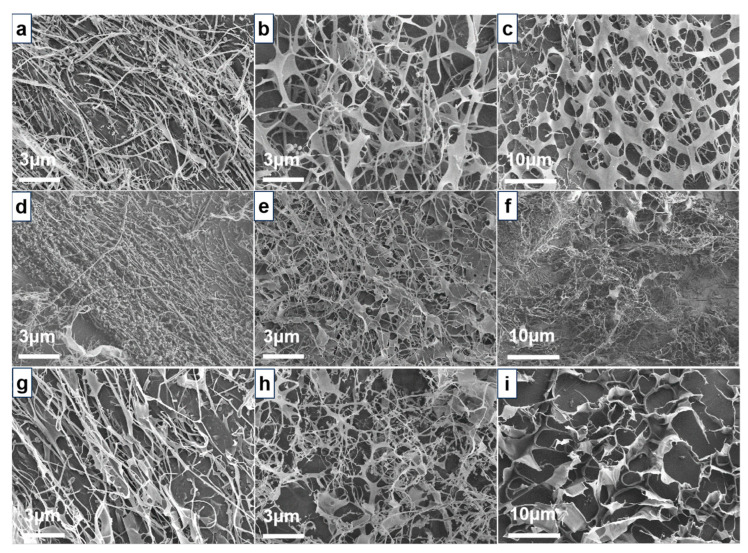
SEM images of polymers at pH 7 with different concentrations: (**a**–**c**) HPAAB at 0.5, 0.8, and 2.0 g/L, respectively; (**d**–**f**) HPAAF at 0.5, 1.0, and 2.0 g/L, respectively; (**g**–**i**) HPAAS at 0.5, 1.3, and 2.0 g/L, respectively.

**Figure 7:**
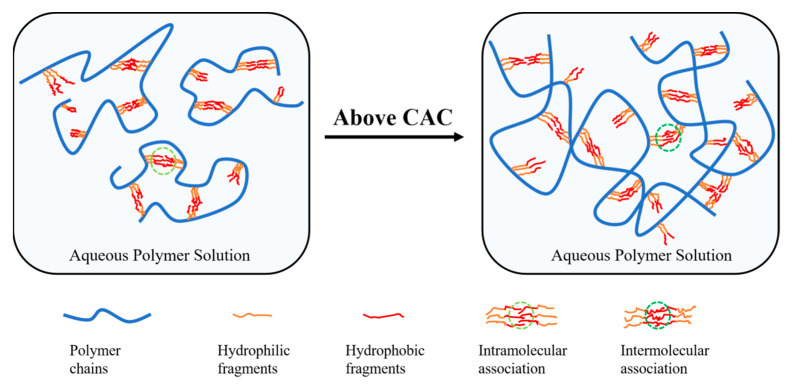
Schematic diagram of the thickening mechanism for hydrophobic associating polymers.

In order to clarify the hydrophobic associating process of HAPAM polymers, SEM images of the polymer before and after the CAC point were investigated (Figure 6). All polymers exhibit filamentous structures and are mainly composed of intramolecular associations at a low concentration of 0.5 g/L. Upon reaching the concentration of the CAC point, the three-dimensional network structure of HPAAB (0.8 g/L) is more uniform and dense compared to that of HPAAF (1.0 g/L) and HPAAS (1.3 g/L). The BEM in HPAAB contains 25 segments of polyethylene glycol and 22 carbon alkyl chains; its longer molecular chain and strong hydrophobic interactions facilitate intermolecular associations, contributing to the formation of stable polymer frameworks. When the concentration of the polymer increases to 2 g/L, the framework of the HPAAF resembles an irregular honeycomb structure, while the framework of the HPAAB remains uniform and stable. The schematic diagram illustrating the hydrophobically associating process is presented in Figure 7.

To further clarify the role of hydrophilic and hydrophobic fragments in the hydrophobic monomers of HPAAB, we synthesized the reference polymer HPAAD using the micellar polymerization method (Figures S6 and S7). The main difference between HPAAB and HPAAD is that the latter uses the completely hydrophobic behenyl acrylate as the hydrophobic chain. The CAC of HPAAD (1.27 g/L) is higher (Figure S8) and the network formed in the solution is irregular (Figure S9). This indicates that the polyethylene glycol units enhance the intermolecular hydrophobic association, resulting in a more uniform network structure. The formation of a network structure in the polymer solution can be visualized dynamically by SEM at different concentrations, which is helpful in studying the association mechanism of polymer systems [46].

#### 2.3.3. Effect of pH on Viscosity

**Figure 8:**
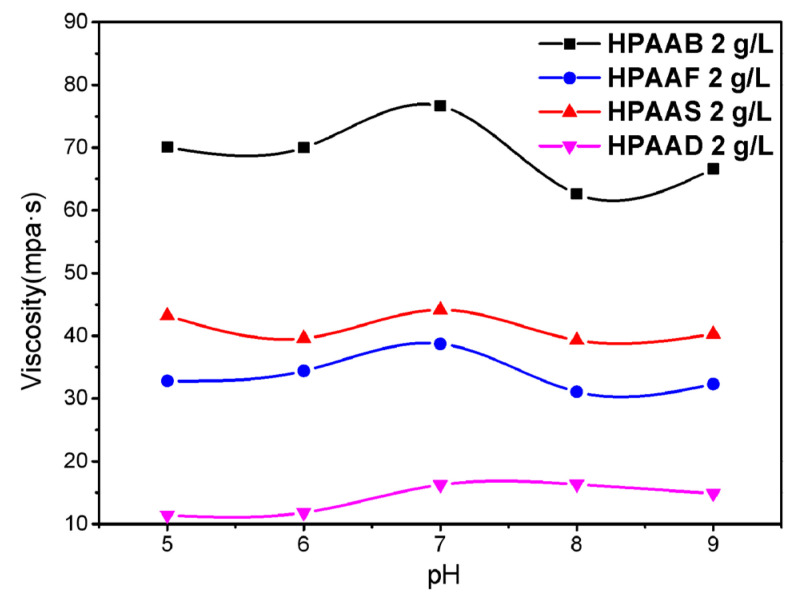
The effect of pH on the thickening ability of HPAAB, HPAAF, HPAAS, and HPAAD.

**Figure 9:**
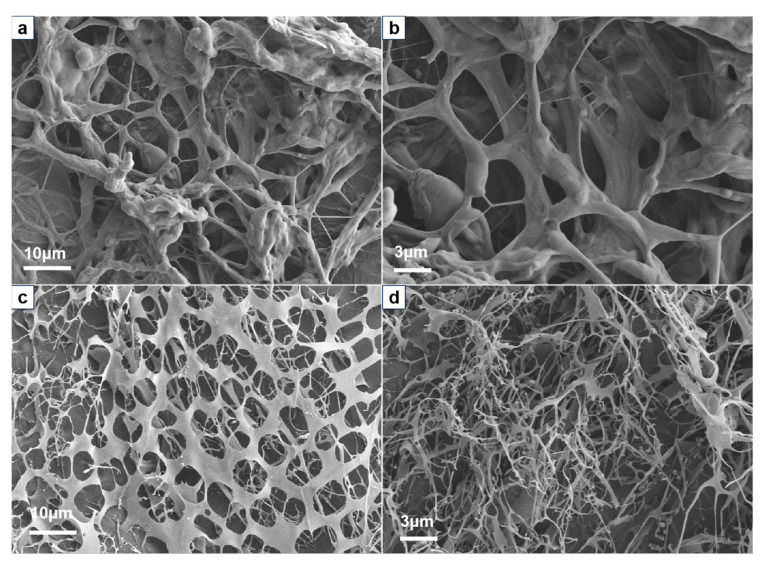
SEM images of HPAAB (2.0 g/L) at different pH values: (**a**) pH = 5; (**b**) pH = 6; (**c**) pH = 7; (**d**) pH = 9.

The effect of pH on viscosity in polymer solutions is shown in Figure 8. The apparent viscosity was measured at a concentration of 2 g/L, ensuring that all polymers formed an effective network structure. The viscosity retention rates of HPAAB, HPAAF, and HPAAS in solution remained at 82%, 80%, and 89%, respectively, within the pH range of 5–9, indicating that HAPAMs have good thickening ability. The maximum viscosities of all polymers were achieved at pH 7, suggesting that the polymer network structures were well-formed in neutral environments. The viscosity of HPAAB in acidic (pH = 5 and 6) and alkaline (pH = 9) solutions was much lower than in a neutral solution (pH = 7); the SEM images indicated that the associating network was irregular in an acidic solution (Figure 9a,b) and partial intramolecular associations began to form in an alkaline solution (Figure 9d). Therefore, the strong intermolecular association and stable three-dimensional structure formed by polymers in solution played a role in enhancing the viscosity of HAPAM systems.

#### 2.3.4. Effect of Temperature on Viscosity

**Figure 10:**
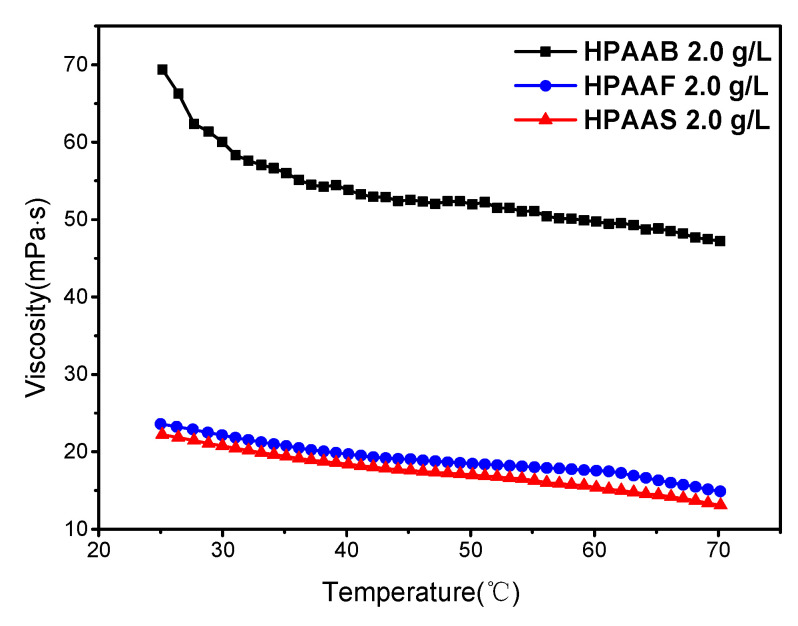
The effect of temperature on the thickening ability of HPAAB, HPAAF, and HPAAS.

The thickening ability of polymer solutions at temperatures ranging from 25 to 70 °C is shown in Figure 10. Generally, the viscosity of the three polymers decreased with increasing temperature. However, the variations in the viscosity of HPAAB, HPAAF, and HPAAS were minimal within the temperature range of 40–50 °C, with changes of 3.4%, 6.3%, and 7.5%, respectively. The thermal motion of hydrophobic groups and water molecules caused dehydration of the polymer; the polymer chains were curled, and the hydrophobic associating effects were weakened, resulting in viscosity reduction at high temperatures [47]. However, hydrophobic interaction is also an entropy-driven process, and an increase in temperature will enhance hydrophobic binding and obtain higher viscosity [48]. Thus, the viscosity was kept relatively stable in the range of 40–50 °C. The hydrophilic units of the BEM monomer in HPAAB also played a vital role in its thickening ability. Without the 25 polyethylene glycol units, HPAAD exhibited a viscosity of 9.6 mPa·s at 50 °C (Figure S10), while HPAAB maintained a viscosity of 51.9 mPa·s at the same temperature. Meanwhile, HPAAF and HPAAS contained a fluorocarbon chain and benzene ring structure, respectively. Their steric hindrance effects on the hydrophobic unit were low, giving viscosities of 18.5 and 17.0 mPa·s at 50 °C, respectively. The longer hydrophobic chain and stronger hydrophobic effect of the BEM group in HPAAB showed significant improvements in temperature tolerance.

#### 2.3.5. Shear Stability

**Figure 11:**
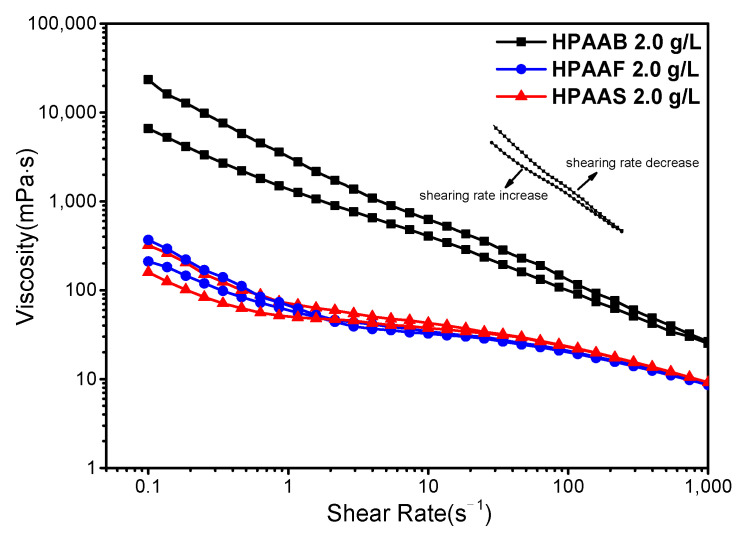
The effect of shear rate on the thickening abilities of HPAAB, HPAAF, and HPAAS.

The rheological curves of the polymer solutions at different shear rates are shown in Figure 11. Each polymer was measured twice, first at a shear rate ranging from 0.1 to 1000 s^−1^ and then again from 1000 to 0.1 s^−1^. All polymers exhibited shear thinning behavior as the shear rate increased. The viscosities of HPAAB and HPAAS remained relatively stable within the shear rate range of 1–100 s^−1^. At high shear rates, the intermolecular association was weakened, and the network of polymer chains was disrupted, leading to viscosity loss in the polymer solution. However, the C-F bonds of HPAAF and delocalized π bonds of HPAAS were strongly hydrophobic chains with a smaller spatial resistance, resulting in rapid dissociation and re-association equilibria. Meanwhile, a similar phenomenon was observed in the rheological curve of HPAAD (Figure S11). The viscosity of the HPAAB polymer showed an approximately linear relationship with the shear rate, likely due to the steric hindrance effect of the large hydrophobic chains. Of significant note, under the same shear rate, the viscosity of HPAAB was significantly higher than that of HPAAF and HPAAS, indicating a stronger hydrophobic association in the polymer chains of HPAAB. Given the viscosity properties of the HPAAB polymer under different shear conditions, we selected it for use in the digital ink-jet printing process since it, which has good thickening ability and strong shear requirements.

### 2.4. Printability of the Aqueous Inks

In conclusion, HPAAB is a promising thickener with excellent thickening capabilities across varying pH, temperature, and shear rate values. Therefore, we applied it in water-based ink and compared its performance with the commercial thickeners TT-935 and RM2020NPR. In the inkjet printing process, the physical properties of aqueous ink, such as its viscosity and surface tension, significantly influence the jetting behavior of the ink droplets. High viscosity and surface tension can increase the injection pressure, while low viscosity and surface tension cause undesirable ink dripping and spreading behavior over the print head nozzle [49,50].

Therefore, the jetting behavior of the fluid can be determined using the Ohnesorge number (*Oh*) of the ink. The inverse values of the Ohnesorge number (*Z*) for the aqueous inks were determined according to the following Equation (1):

(1) $$Z=Oh^{-1}=\frac{\sqrt{\rho \gamma L}}{\eta }$$

where *η* is the viscosity (mPa·s), *ρ* is the density (kg/m^3^), *γ* is the surface tension (mN/m), and *L* is the characteristic length scale, which is equal to the inkjet orifice diameter [51]. It was suggested that a *Z*-value of ink within the range of 1 and 10 should be recommended for suitable jetting behavior and stable drop formation. The physical properties and parameters of aqueous ink are listed in Table 1.

The *Z*-value of the reference aqueous ink was 11.67, due to the low viscosity and high surface tension of water, which results in both the lengthening of the droplet tail and the generation of satellite droplets [52]. With the addition of thickeners, the *Z*-value gradually decreased as the viscosity of the ink increased. The addition of 0.1 wt.% HPAAB obtained a *Z*-value of 5.96, which is the most suitable value for the inkjet printing process [53]. The reduction of surface tension and the enhancement of viscosity resulted in a proper *Z*-value for the aqueous ink. Meanwhile, the addition of 0.15 wt.% TT-935 also yielded a *Z*-value of 5.64. Under the same formulation, HPAAB proved to be more efficient than the TT-935 thickener. However, after adding 0.1–0.5 wt.% of RM2020NPR, the *Z*-value remained high due to the slow increase in the viscosity of aqueous ink.

**Figure 12:**
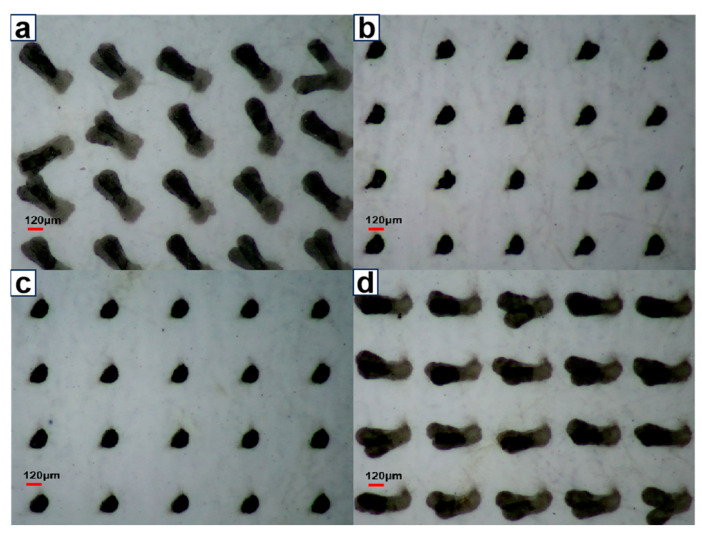
The inkjet-printed dot images of the aqueous ink containing different thickeners: (**a**) reference ink; (**b**) 0.1 wt.% HPAAB; (**c**) 0.1 wt.% TT-935; (**d**) 0.1 wt.% RM2020NPR.

The jetting behavior and printability of the various aqueous inks with different thickeners are displayed in Figure 12. Without thickener being added to the reference ink, the printed dots exhibited long droplet tails. A similar tailing phenomenon was observed in aqueous ink containing RM2020NPR thickener, which was attributed to its low viscosity. In contrast, the ink with HPAAB and TT-935 had suitable viscosity and *Z*-values, resulting in clear contours in the printed dots, with diameters concentrated between 100 and 130 μm. As shown in Figure S12, the printed line contours of the HPAAB and TT-935 inks were also much clearer and finer than the reference ink and TT-935 ink. Thus, the rheological properties of aqueous inks significantly influenced their jettability in the inkjet printing process.

### 2.5. Stability of the Aqueous Inks

**Figure 13:**
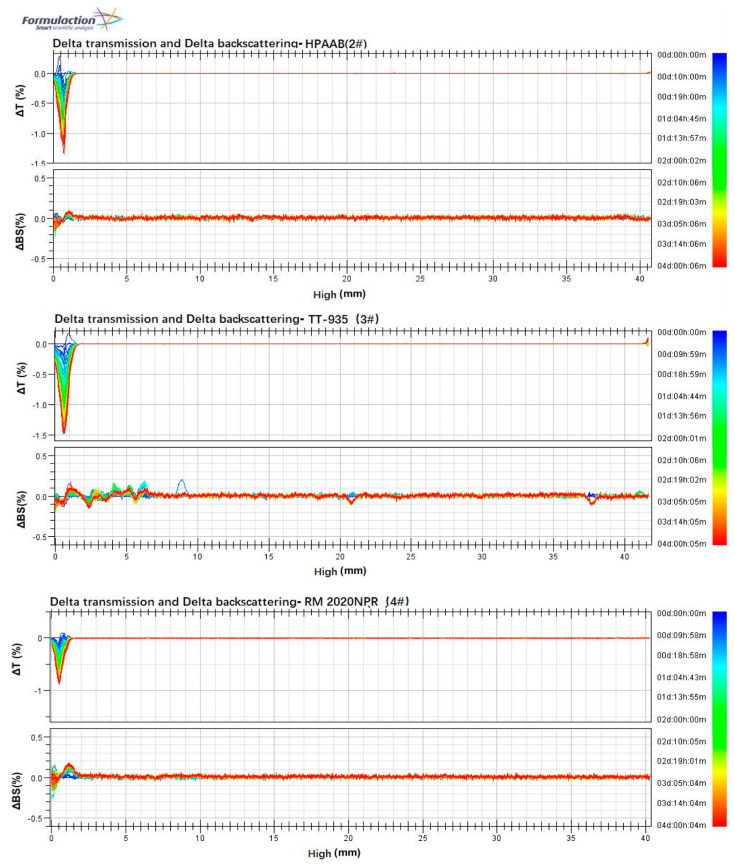
Turbiscan spectra of the thickeners in aqueous ink at room temperature: (**a**) 0.1 wt.% HPAAB (2# ink); (**b**) 0.1 wt.% TT-935 (3# ink); (**c**) 0.1 wt.% RM2020NPR (4# ink).

The stability of aqueous ink was evaluated using a multiple light-scattering instrument, which analyzes particle movements and polymer configurations in concentrated solutions by measuring transmission and backscattering over time and according to sample position [54]. The aqueous ink with 0.1 wt.% HPAAB thickener showed significant stability and no obvious precipitation on the bottom of the tube after 4 days of measurements (Figure 13). The variations in backscattering (ΔBS) and transmission (ΔT) for HPAAB were minimal, indicating that a stable polymer network had formed in the ink. However, as the temperature or the contents of thickeners in the ink increased, the stability of all the inks tended to decline (Table S1).

## 3. Experimental Section

### 3.1. Materials

The materials used in this study were purchased from the following suppliers: AM and AMPS were purchased from Aladdin Scientific Corp., Shanghai, China; SDS was purchased from Jiuding Chemical (Shanghai) Technology Co., Ltd., Shanghai, China; BEM was purchased from Zhangjiagang Renda Chemical Co., Ltd., Suzhou, China; FEA and ST were purchased from Guangzhou Rui Shi Biotechnology Co., Ltd., Guangzhou, China; Pyrene and potassium persulfate were purchased from Macklin Biotech Co., Ltd., Shanghai, China; TT-935 and RM-2020NPR were purchased from Rohm & Haas, Philadelphia, USA. TT-935 is a hydrophobically modified anionic thickener and alkali swellable emulsion, while RM-2020NPR is a non-ionic, solvent-free, hydrophobically modified polyurethane rheological modifier. All reagents were obtained from commercial sources and were used without further purification. The deionized water used in this study was produced in-house in the laboratory.

### 3.2. Preparation of Copolymers

The copolymers of AM, AMPS, and BEM/FEA/ST were prepared by micellar copolymerization, using SDS as the surfactant and potassium persulfate as the redox free radical initiator. Each reaction was conducted in a 1000 mL, four-necked, round flask equipped with a mechanical stirrer and a nitrogen inlet and outlet. AM and AMPS were added to the reaction flask and dissolved in deionized water, respectively. Sodium hydroxide was added to adjust the pH to 6–7, followed by the addition of SDS. The hydrophobic monomer was added, and the mixture was stirred under a N_2_ atmosphere until a clear, homogeneous mixture was observed. The total monomer concentration in the water was maintained at 20% (*w*/*w*) with an initiator concentration of 0.6% (*w*/*w*), based on the total amount of monomer. After the addition of the initiator, the polymerization proceeded at 55 °C for 4 h to obtain a gel polymer. The obtained polymers were washed with acetone and extracted with ethanol for 8 h to remove any traces of water, surfactant, and residual monomer. All samples were dried at 60 °C under vacuum until a constant weight was achieved.

### 3.3. Measurements

FTIR analyses were performed with a Bruker TENSOR II spectrum (Berlin, Germany) using KBr pellets. The ^1^H-NMR, ^13^C-NMR, and ^19^F-NMR spectra were obtained with a Bruker AVANCE NEO 600 nuclear magnetic resonance spectrometer (Zurich, Switzerland), using D_2_O as the solvent at room temperature. SEM imaging was performed with a Verios 5UC (Thermofisher, Waltham, USA) and Gemini 450 (Cambourne, Zeiss, UK). The polymer solution samples were dried in a freeze-dryer before observing their morphology. Fluorescence spectrometry (QM/TM, PTI, San Jose, USA) was performed with pyrene as a fluorescent probe at a concentration of 5 × 10^−6^ g L^−1^; the range of the emission wavelength was 350–550 nm, the excitation wavelength was 335 nm, the emission slit width was 2 nm, and the step size was 1 nm. The apparent viscosity was determined with an NDJ-5S digital rotational viscometer (Lichen, Shanghai, China) with rotor No. 1 (60 rpm) at 25.0 ± 0.1 °C. All samples needed to be stirred thoroughly before conducting viscosity testing. The rheological properties of the polymers were studied using an MCR702e rotational rheometer (Anton Paar, Graz, Austria) with the cone-plate CP40-1 at 30.0 ± 0.1 °C, along with a shear rate of 60 s^−1^ for analyzing temperature effects on viscosity. A surface tension analyzer (Sigma 700, Biolin, Espoo, Finland) was used to measure the surface tension of the aqueous ink. The sedimentation behavior and dispersion stability of the pigment particles and polymer conformation in the aqueous ink were analyzed quantitatively using a multiple light scattering instrument (TURBISCAN TOWER, Formulaction, Toulouse, France). The printability of the aqueous ink was determined using an aerosol jet system (200 System, Optomec, Albuquerque, USA), which had a pneumatic ink-jet print head with a nozzle 300 μm in diameter. The current of the ultrasonic atomizer in the print head was 0.449 A. The ink output of each inkjet printing point was 60 standard cubic centimeters per minute (SCCM), with a pressure of 1.36 PSI. The ink output of each inkjet-printing line pattern was 30 SCCM, with a pressure of 0.68 PSI. The aqueous ink was printed on a white board paper substrate, with dot-to-dot and line-to-line distances of 500 μm and 1000 μm, respectively.

### 3.4. Determination of Viscosity-Average Molecular Weight

The viscosity-average molecular weight was determined by the viscosity method according to GB/T 17514-2017 [55]. According to the reported research work, the one-point method should be used to determine the intrinsic viscosity [*η*] of polymers [21,56,57]. The [*η*] of the polymers was determined using an Ubbelohde viscometer (4-0.57, Taizhou Jiaojiang Huanguang Glass Instrument Co., Taizhou, China) in 1 mol L^−1^ NaCl solution, with the temperature maintained at 30 ± 0.5 °C. The [*η*] value was calculated using Equation (2):

(2) $$\left[\eta \right]=\sqrt{2\left(\eta_{sp}-\ln\eta_{r}\right)}$$

where *η_r_* stands for the relative viscosity calculated from Equation (3) and *c* stands for the concentration of polymer solution. Where *t* and *t*_0_ are the time that the polymer solution and the 1 mol L^−1^ NaCl solution spent flowing through the upper and lower scale lines of the Ubbelohde viscometer, respectively:

(3) $$\eta_{r}=t∕t_{0}$$

where *η_sp_* stands for the specific viscosity, calculated from Equation (4).

(4) $$\eta_{s_{P}}=\left(t∕t_{0}\right)-1$$

where *M* stands for the viscosity-average molecular weight, calculated with the Mark–Houwink equation (Equation (5)), and *K* and *α* stand for the empirical constants related to temperature and solvent during measurement. The *K* value of polyacrylamide in NaCl solution is 4.75 × 10^−3^, and the *α* value is 0.8.

(5) M=η∕K1α

### 3.5. Preparation of the Aqueous Ink

The aqueous digital ink samples were formulated by mixing them with 8 wt.% of black water-based color paste (LUNAJET B-K, Kao Corp., Tokyo, Japan), 30 wt.% of propylene glycol, 0.5 wt.% of surfactant (SAFOL EN90, Sasol Chemical Co., Nanjing, China), 4 wt.% of waterborne acrylic resin, deionized water, and a thickener (HPAAB, TT-935 or RM2020NPR) at the content of 0.1–0.5 wt.%. The water-based ink samples were kept under stirring for 30 min at room temperature until well dispersed.

## 4. Conclusions

In summary, we have designed and synthesized three novel hydrophobically modified polyacrylamides, namely, HPAAB, HPAAF, and HPAAS, which incorporate the following molecular engineering concepts: (1) the mixed hydrophilic and hydrophobic fragments in the hydrophobic BEM of HPAAB contribute to enhancing the intermolecular hydrophobic association and making the hydrophobic network structure more uniform. (2) Compared with HPAAF and HPAAS, the longer hydrophobic chain and stronger hydrophilic–hydrophobic interactions of the BEM group in HPAAB showed significant thickening ability across various pH, temperature, and shear rate values. (3) As a thickener in aqueous ink, HPAAB exhibited superior printability and stability compared to the corresponding commercial thickeners, which may be due to a stronger hydrophobic associating effect and a stable network structure in the ink system. To the best of our knowledge, this is the first instance of achieving enhanced printability and stability in aqueous ink using hydrophobically modified polyacrylamides that feature both hydrophilic and hydrophobic units in hydrophobic side chain. These findings present new opportunities for developing efficient and cost-effective thickening agents in aqueous ink for digital inkjet printing.

## Figures and Tables

**Table 1:** The physical properties and parameters of aqueous ink.

Thickener	Content (wt.%)	Viscosity (mPa·s, *η*)	Surface Tension (mN/m, *γ*)	Inverse of Ohnesorge Number, *Z*
Reference ink	0	4.12	36.18	11.67
HPAAB (2# ink)	0.1	8.15	35.65	5.96
	0.15	8.36	35.15	5.69
	0.2	9.57	34.98	4.93
	0.3	12.5	34.37	3.75
	0.5	20.7	34.29	2.24
TT-935 (3# ink)	0.1	6.46	34.34	7.23
	0.15	8.23	34.02	5.64
	0.2	10.27	32.8	4.44
	0.3	13.47	33.46	3.37
	0.5	25.4	33.06	1.77
RM2020NPR (4# ink)	0.1	4.32	34.47	10.82
	0.15	4.93	34.37	9.48
	0.2	4.86	34.38	9.61
	0.3	5.21	34.37	8.96
	0.5	6.03	34.31	7.75

## Data Availability

The data that support the findings of this study are available from the corresponding author upon reasonable request.

## Supplementary Materials

The following supporting information can be downloaded at: https://www.mdpi.com/article/10.3390/molecules29215105/s1, Figure S1: The DSC analysis of HPAAB and HPAAS; Figure S2: The NMR spectroscopy of HPAAB; Figure S3: The NMR spectroscopy of HPAAF; Figure S4: The NMR spectroscopy of HPAAS; Figure S5: The viscosity of polymer solutions at different hydrophobic monomer contents; Figure S6: The FTIR spectrum of HPAAD; Figure S7: The NMR spectroscopy of HPAAD; Figure S8: The relationship of concentration with the apparent viscosity of HPAAD; Figure S9: The SEM images of HPAAD at pH 7 with different concentrations: (a), (b), (c), and (d), with 0.5, 0.8, 1.5, and 2.0 g/L, respectively; Figure S10: The effect of temperature on the thickening ability of HPAAD; Figure S11: The effect of the shear rate on the thickening ability of HPAAD; Table S1: The variations in the transmission (ΔT) and backscattering (ΔBS) of inks with different thickeners at 25 and 50 °C; Figure S12: Inkjet-printed line images of the aqueous ink containing different thickeners: (a) reference ink; (b) 0.1 wt.% HPAAB; (c) 0.1 wt.% TT-935; (d) 0.1 wt.% RM2020NPR.
