# Supplementary material for: Hydrophobically Modified Polyacrylamide Incorporating Both Hydrophilic and Hydrophobic Units: Enhanced Printability and Stability in Aqueous Ink
Source: Molecules. 2024 Oct 29;29(21):5105. doi: 10.3390/molecules29215105 (PMC11547646; doi:10.3390/molecules29215105)
Supplement: Supplementary file 1 [file molecules-29-05105-s001.zip › molecules-3262432-supplementary.pdf]

Hydrophobically Modified Polyacrylamide Incorporating both Hydrophilic and Hydrophobic Units: Enhanced Printability and Stability in Aqueous Ink

Zhi-Rui Liu[1,‡], Li-Lin[1] Tan[1,‡,*], Juan Gao[1,2], Zi-Ye Qin[1,2], Xin-Xin Huo[1,2], Zhi-Min Liang[1]

1. Shantou Engineering Technology Research Center for Green and Precise Manufacturing of High-value Chemicals, Chemistry and Chemical Engineering Guangdong Laboratory, Shantou 515031, China; liuzr@ccelab.com.cn (Z.-R. Liu)
2. College of Chemistry and Chemical Engineering, Shantou University, Shantou 515063, China;
* Correspondence: tanll@ccelab.com.cn (L.-L. Tan)
‡ These authors contributed equally to this work.

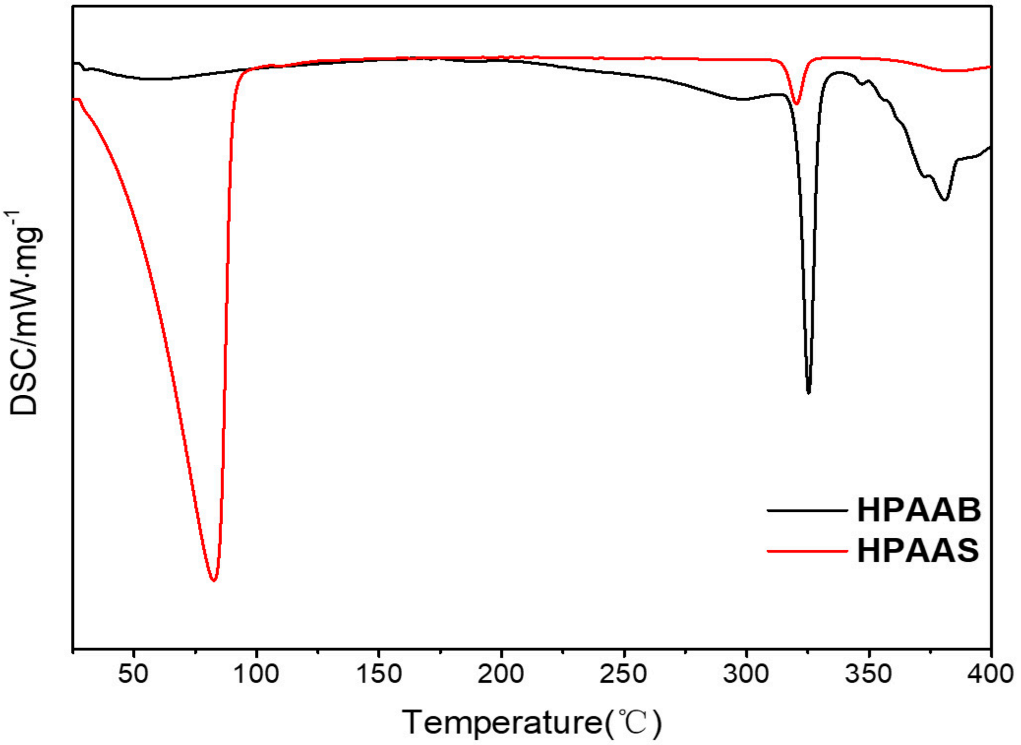

Figure S1. The DSC analysis of **HPAAB** and **HPAAS**.

(a)

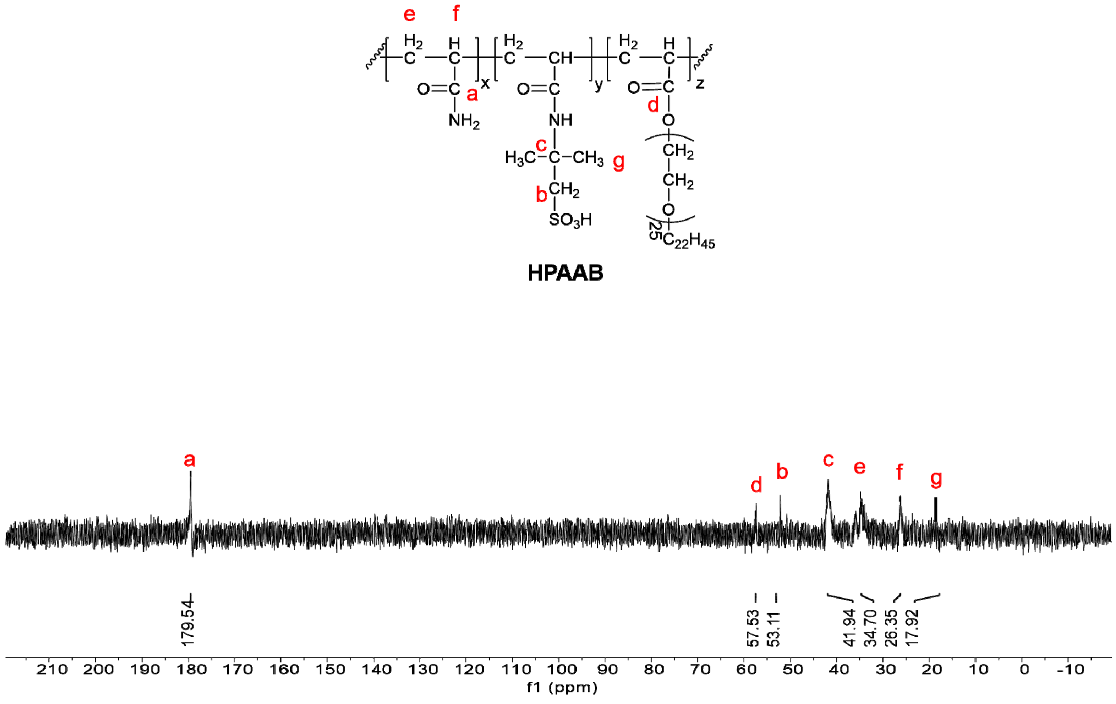

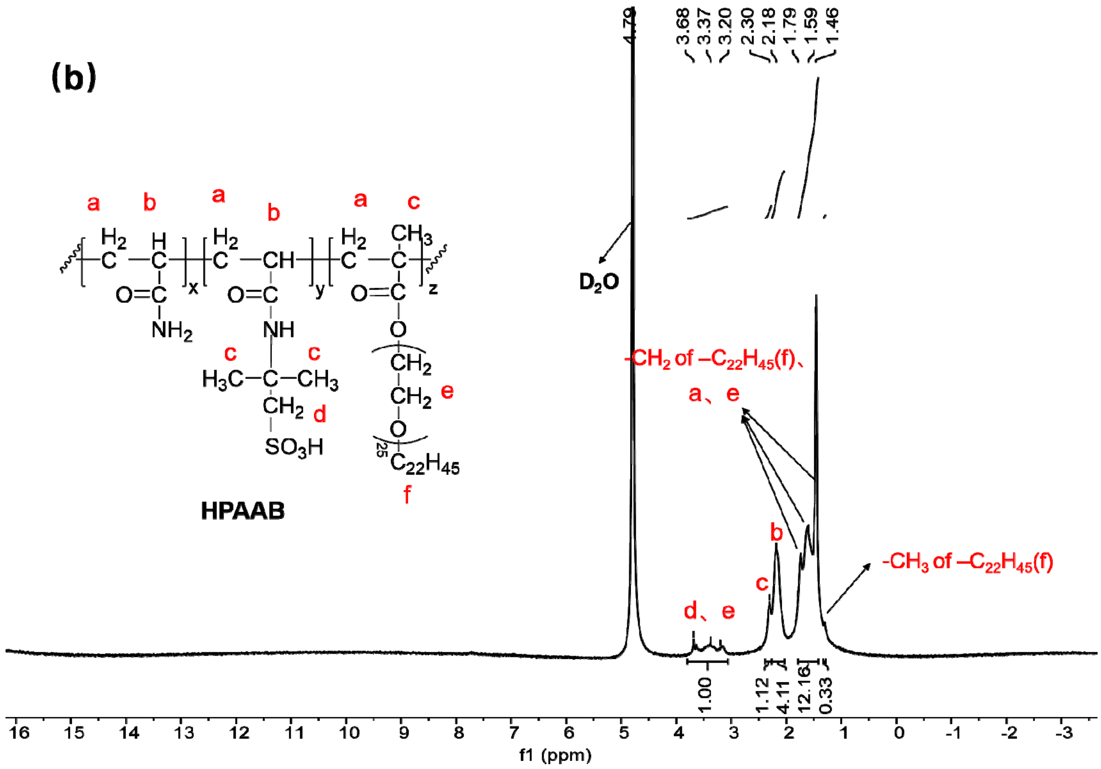

Figure S2. The NMR spectroscopy of **HPAAB**.

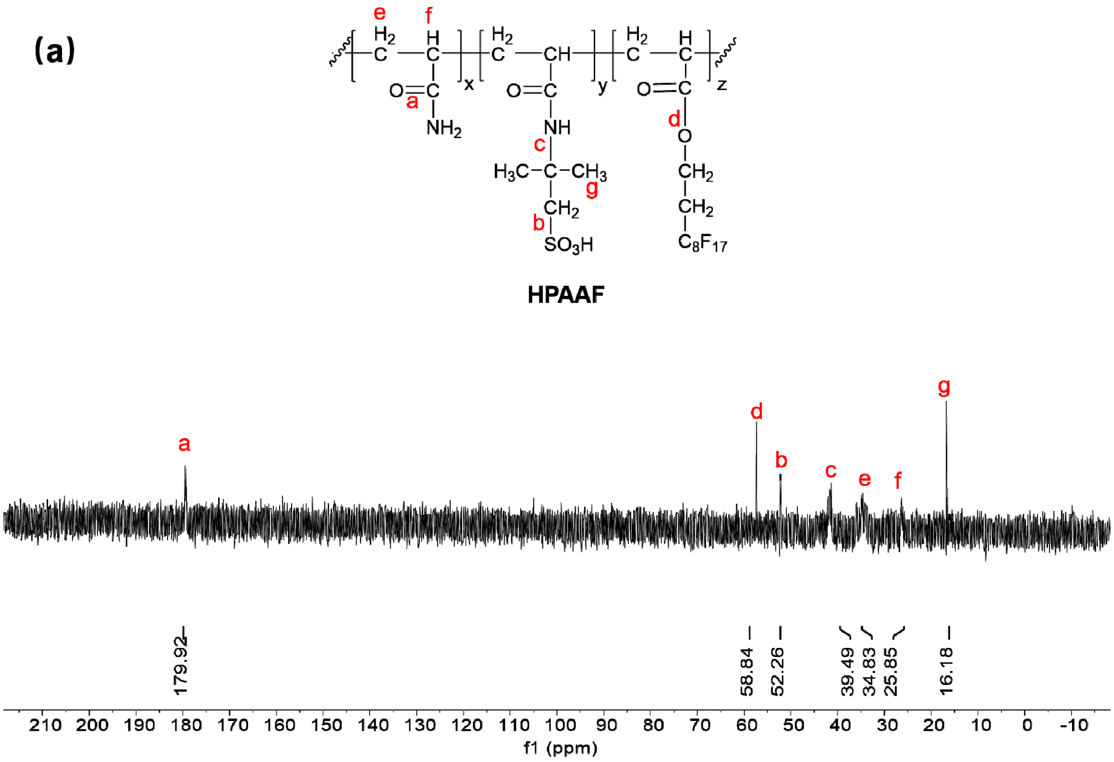

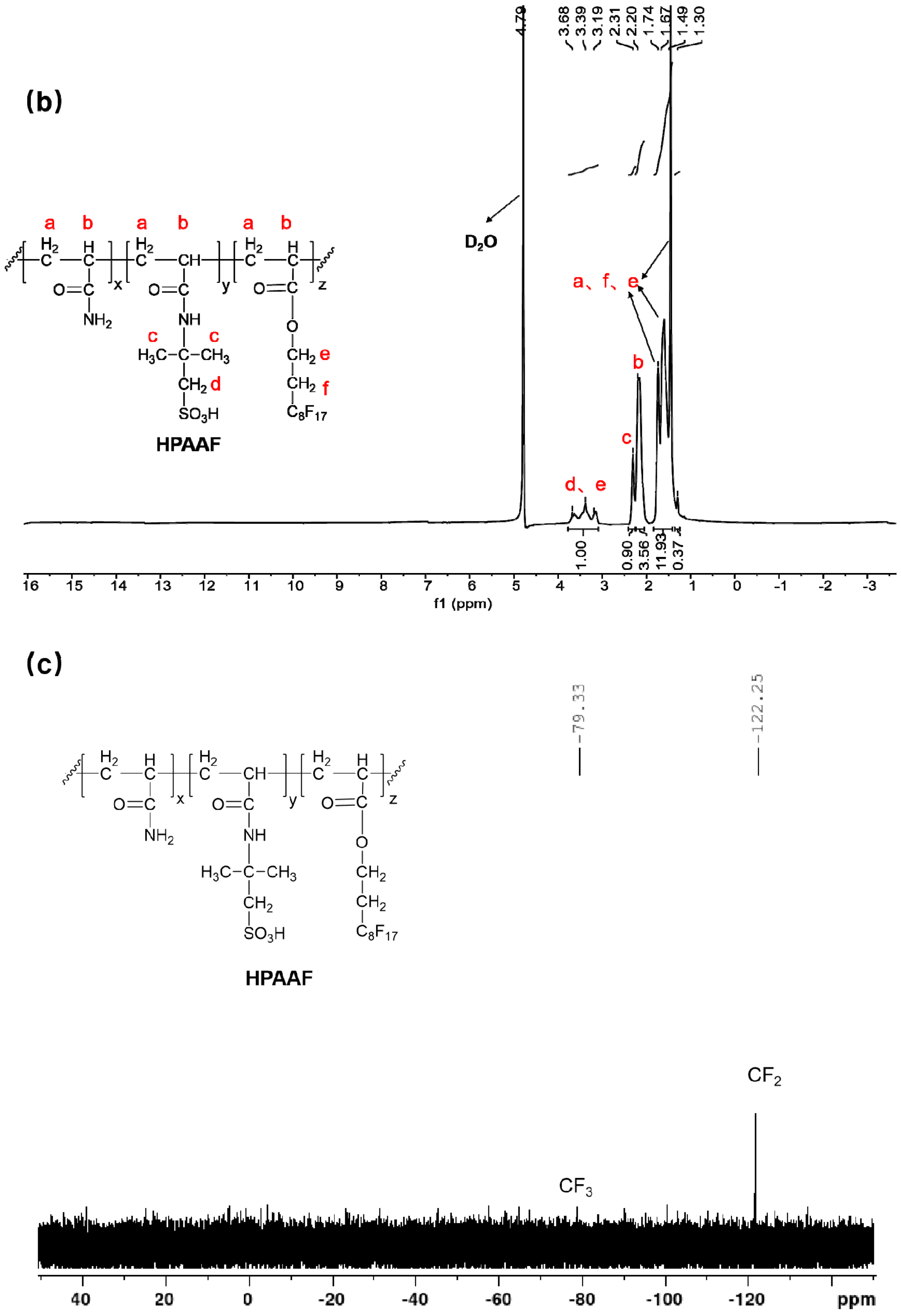

Figure S3. The NMR spectroscopy of **HPAAF**.

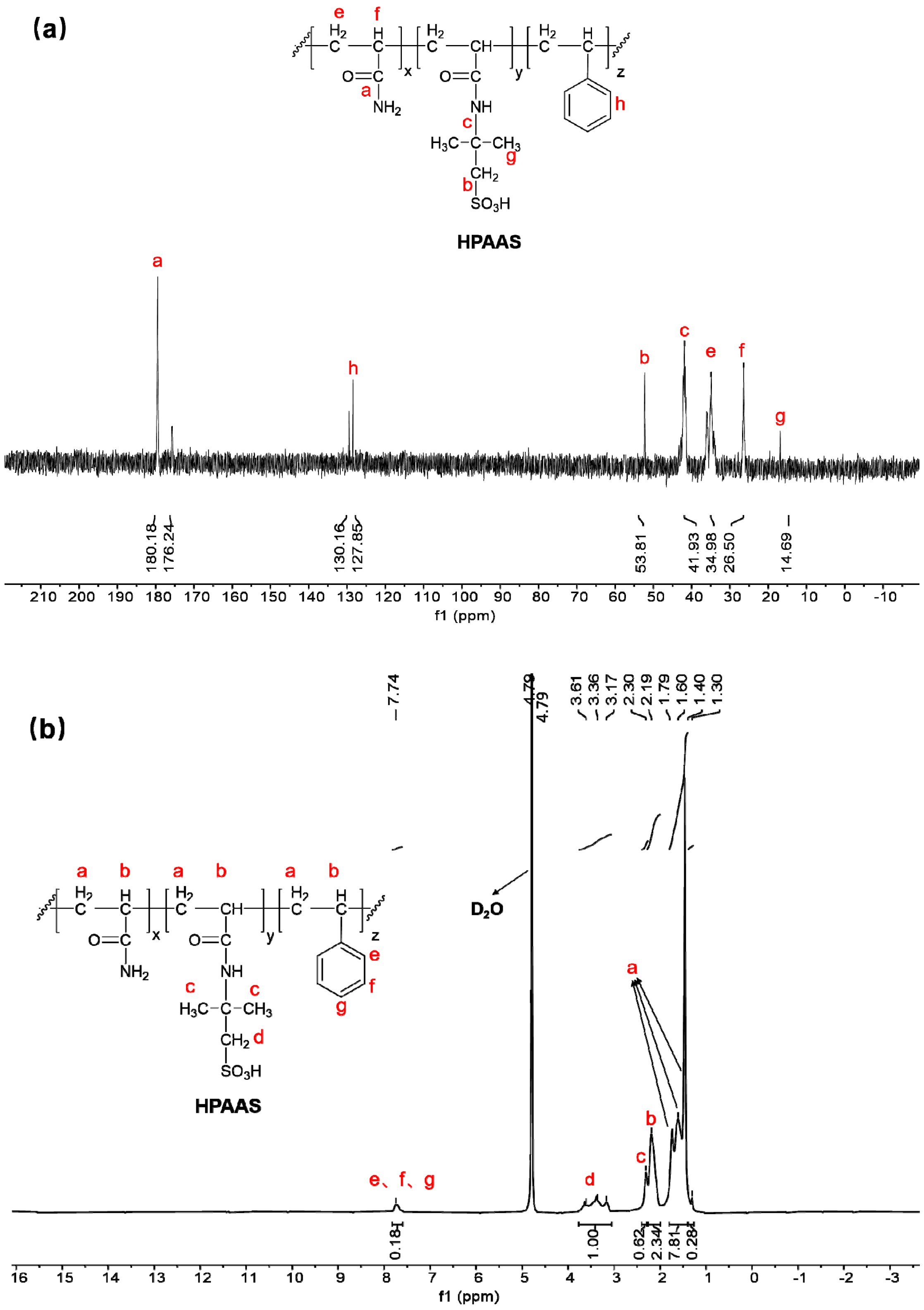

Figure S4. The NMR spectroscopy of **HPAAS**.

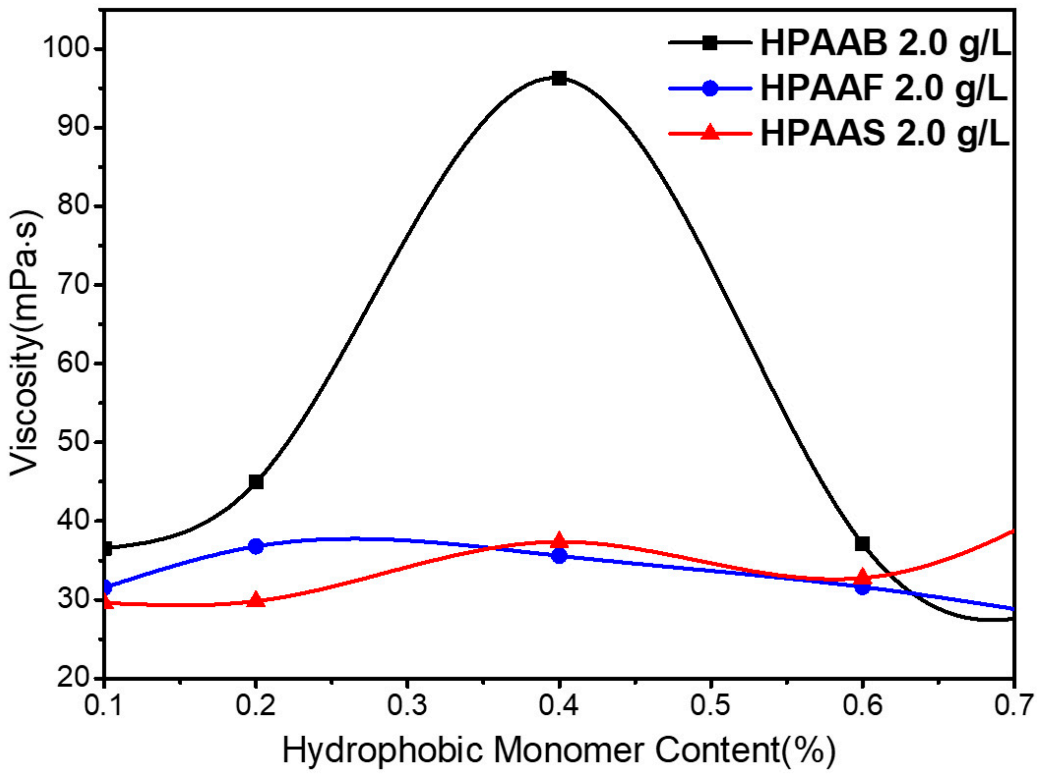

Figure S5. The viscosity of polymer solutions at different hydrophobic monomer contents.

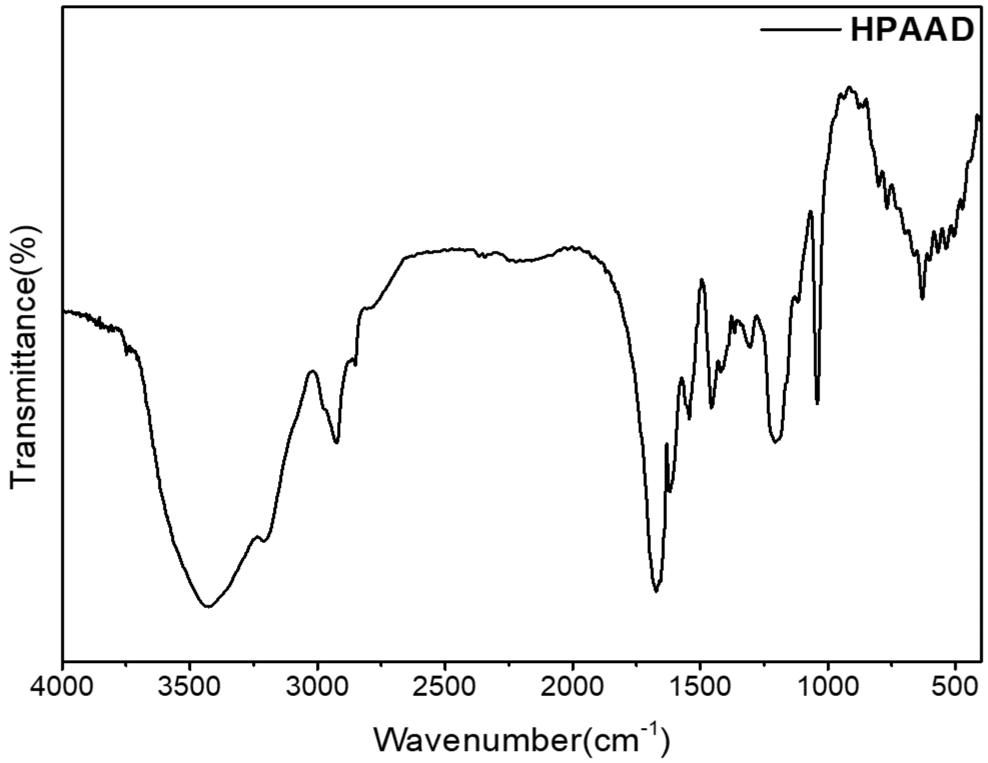

Figure S6. The FTIR spectrum of **HPAAD**.

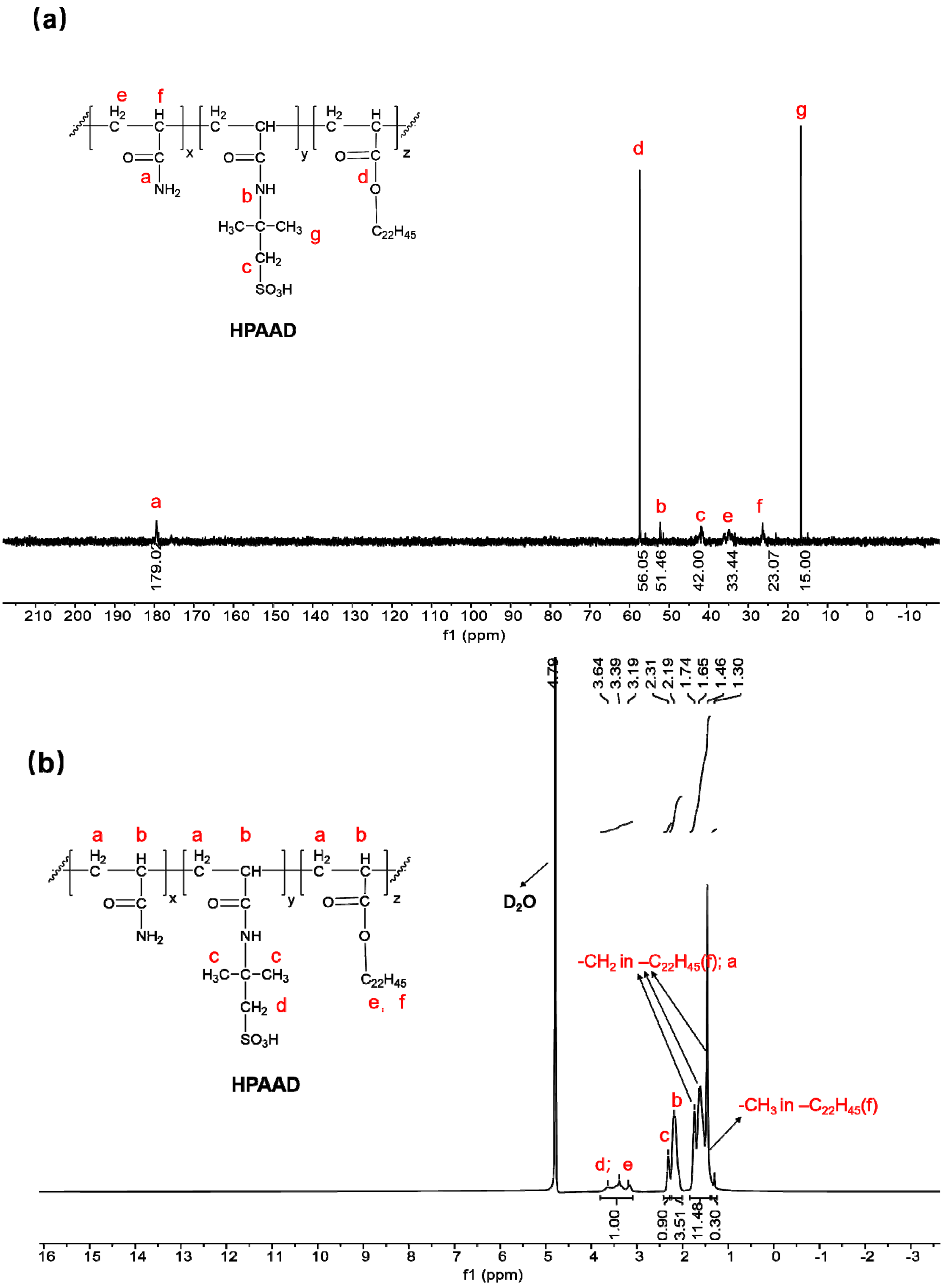

Figure S7. The NMR spectroscopy of **HPAAD**.

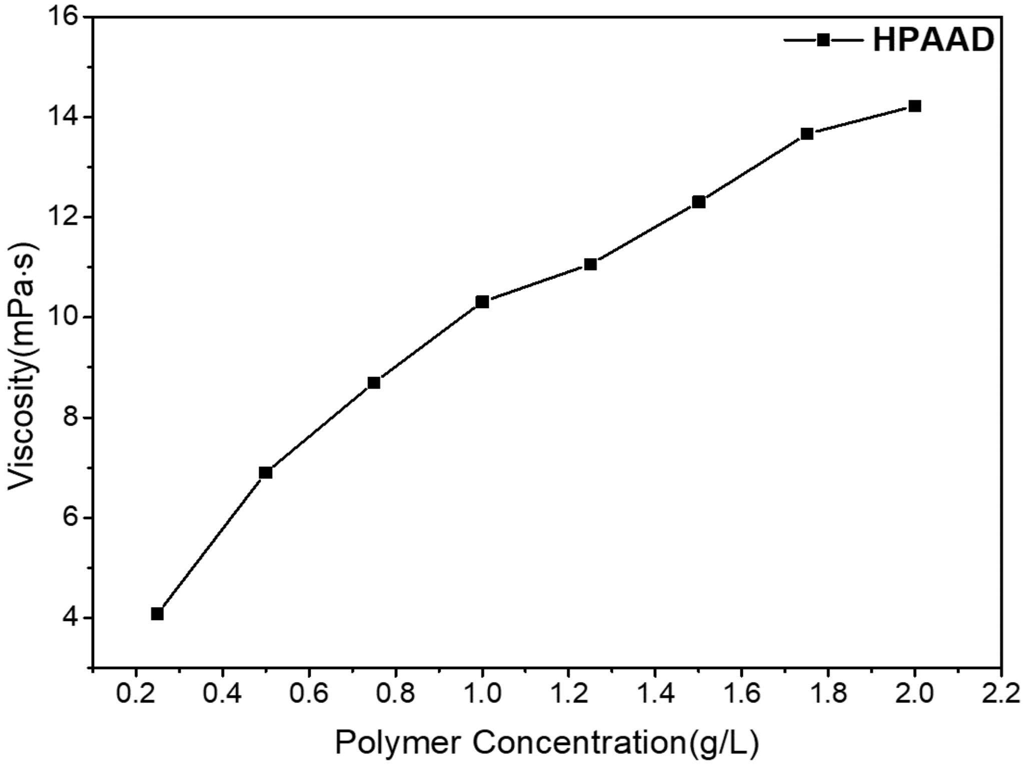

Figure S8. The relationship of concentration with the apparent viscosity of **HPAAD**.

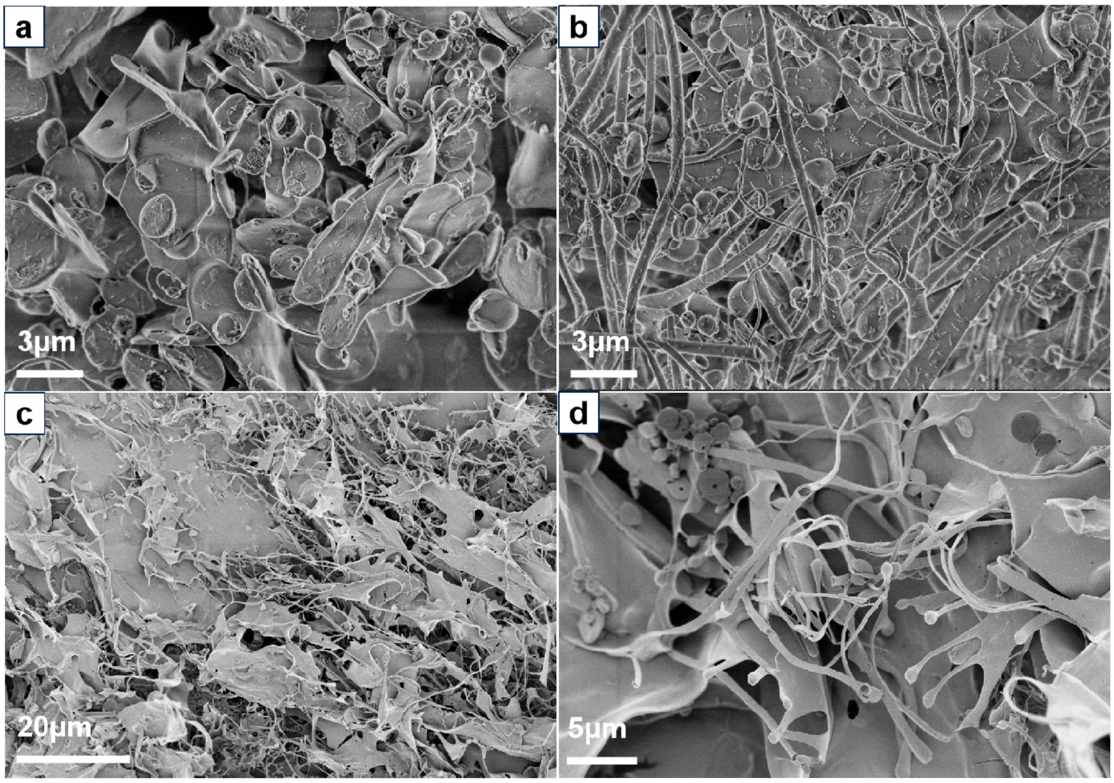

Figure S9. The SEM images of **HPAAD** at pH 7 with different concentrations, (a), (b), (c) and (d), with 0.5, 0.8, 1.5, and 2.0 g/L, respectively.

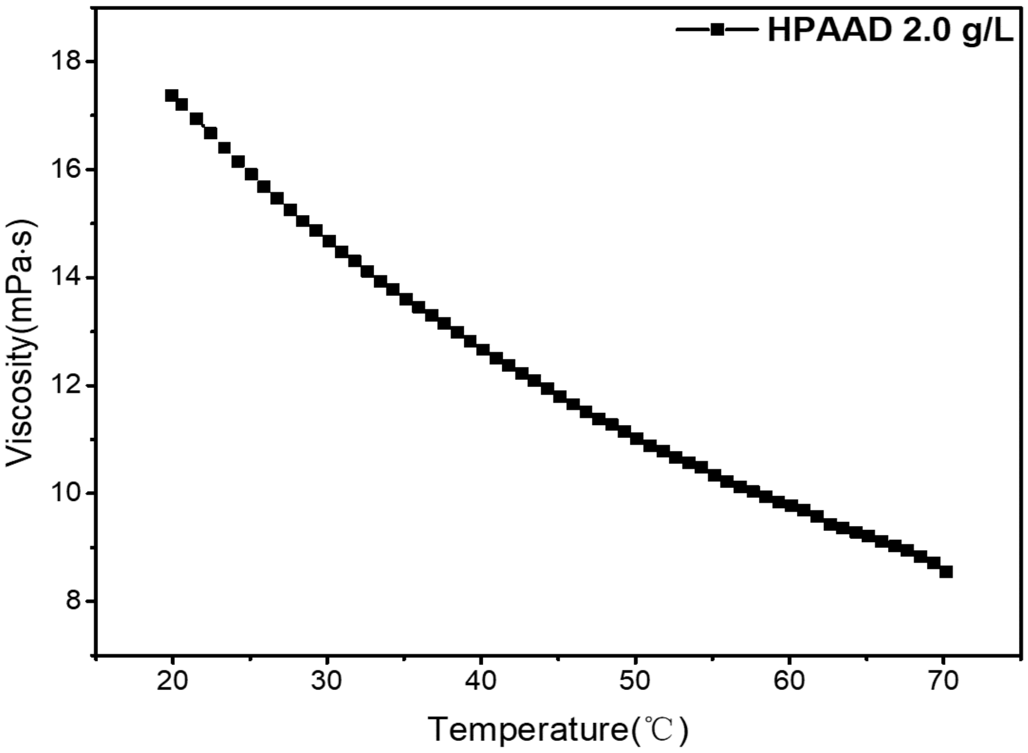

Figure S10. The effect of temperature on the thickening ability of **HPAAD**.

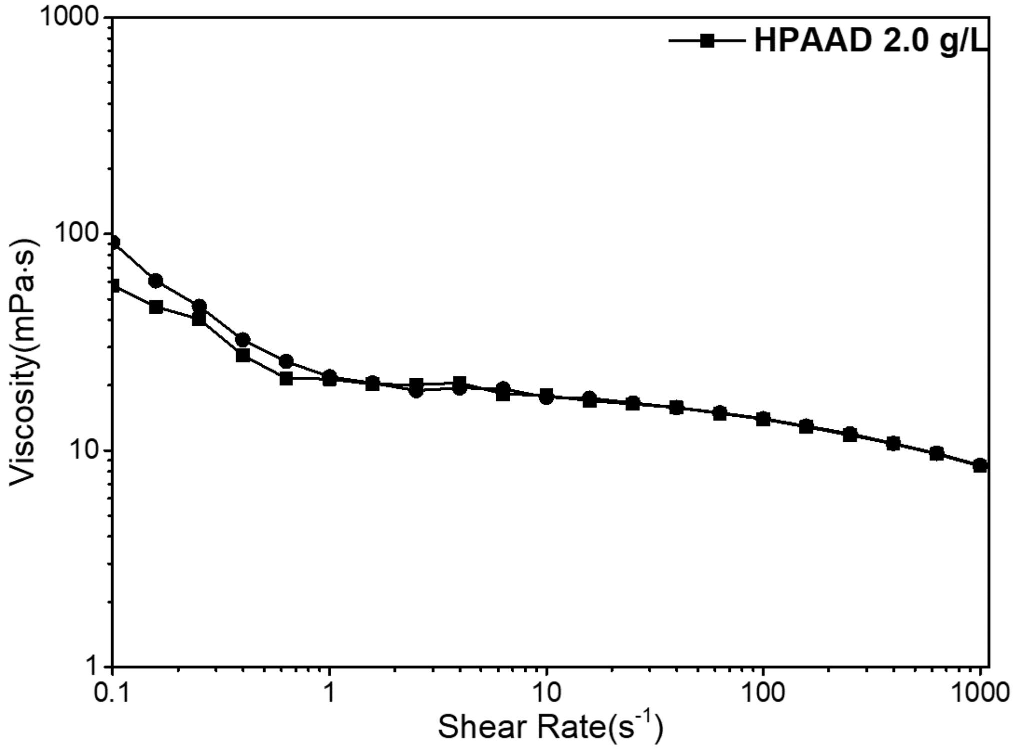

Figure S11. The effect of the shear rate on the thickening ability of **HPAAD**.

Table S1. The variations in the transmission (ΔT) and backscattering (ΔBS) of inks with different thickeners at 25 and 50 ℃.

Thickener	Stability at 25 ℃			Stability at 50 ℃		
	BS at 0 min	ΔT on tube bottom [1] (%)	ΔBS on tube bottom (%)	BS at 0 min	ΔT on tube bottom (%)	ΔBS on tube bottom (%)
**HPAAB**-0.1%（2# ink）	0.743024	-1.32	0.09	0.734500	-0.98	0.11
**HPAAB**-0.15%（2# ink）	0.733448	-2.11	0.23	0.725798	-1.51	0.14
**HPAAB**-0.2%（2# ink）	0.733991	-1.81	0.12	0.748347	-2.19	0.14
TT-935-0.1%（3# ink）	0.767269	-1.51	0.09	0.743384	-2.44	0.15
TT-935-0.15%（3# ink）	0.780123	-2.00	0.2	0.799059	-2.79	-0.20
TT-935-0.3%（3# ink）	0.768162	-3.57	0.11	0.779384	-1.92	0.33
RM2020NPR-0.1%（4# ink）	0.711962	-0.88	0.17	0.705633	-2.23	0.14
RM2020NPR-0.15%（4# ink）	0.727661	-1.57	0.17	0.720751	-2.59	0.21
RM2020NPR-0.2%（4# ink）	0.720318	-4.01	0.27	0.724821	-4.56	0.19

1. There will be a very small amount of precipitation in the gaps between the convex lunar surfaces of the sample tube.

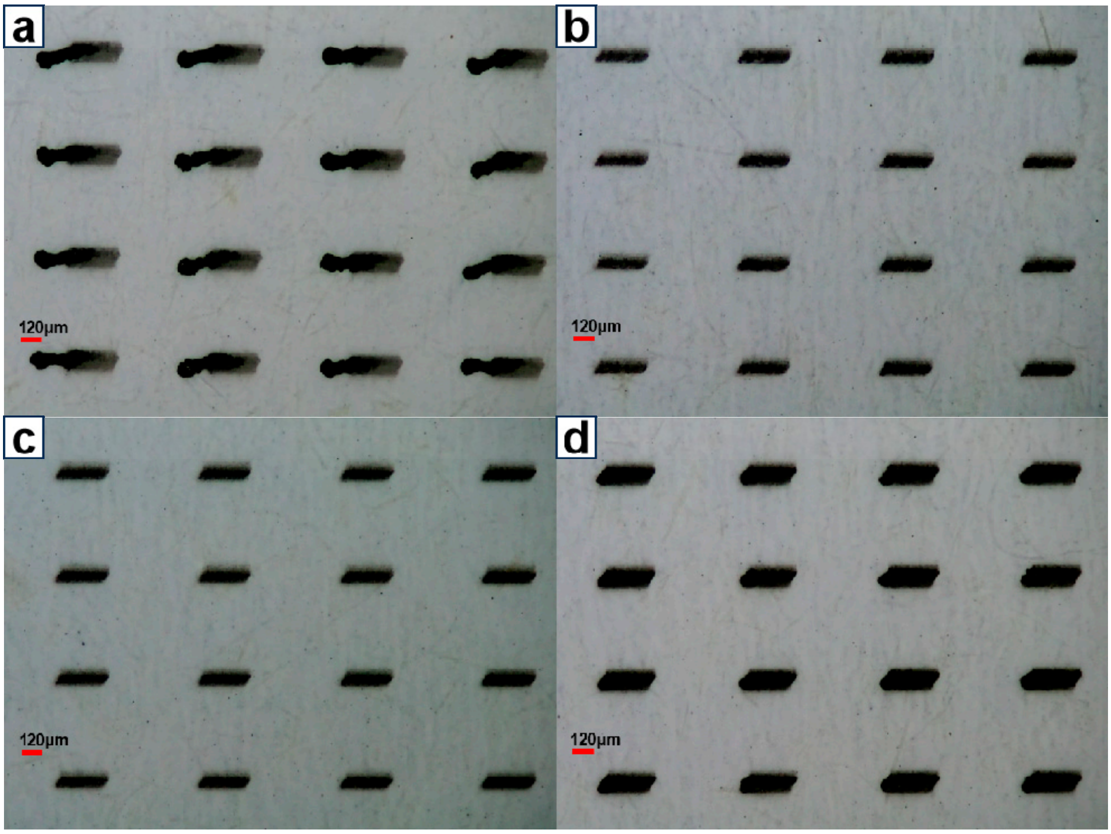

Figure S12. Inkjet-printed line images of the aqueous ink containing different thickeners: (a), reference ink; (b), 0.1wt% **HPAAB**; (c), 0.1wt% TT-935; (d), 0.1wt% RM2020NPR.

Synthesis

Synthesis of HPAAB

AM (9.43 g, 130.0 mmol), AMPS (6.87 g, 33 mmol), deionized water (68.02 g) were added to a four-necked round flask, respectively. Adding NaOH to reaction system and adjust the pH to 6-7, then SDS (1.04 g, 3.60 mmol) was added into reaction flask. The hydrophobic monomer BEM (0.99 g) was added and mixture was stirred under N_2 atmosphere until a clear homogeneous mixture was observed. The initiator potassium persulfate (0.104 g, 0.38 mmol) was added and the total monomer concentration in mixture was kept at 20wt%. The polymerization was conducted at 55°C for 4 h to obtain a gel polymer. The polymer were washed with acetone and extracted with ethanol for several times. The product was dried at 60°C under vacuum for 24 h.

^{1}H-NMR(600 MHz, D_2O) δ (ppm): 3.2-3.68 (d, $-CH_2-SO_3H$ and e), 2.3 (c, $-C-CH_3$), 2.18 (b, -CH-), 1.46-1.79 (a、e、f, $-CH_2-$), 1.49 (f,$-CH_3$); ^{13}C-NMR (150 MHz, D_2O) δ (ppm): 179.54 (a, C=O), 57.53 (d, C-O), 53.11 (b, C-S), 41.94 (c, C-N), 34.70 (e, $-CH_2-$), 26.35 (f,-CH-), 17.92 (g, $-CH_3$).

Synthesis of HPAAF

AM (9.43 g, 130.0 mmol), AMPS (6.87 g, 33.0 mmol), deionized water (65.5 g) were added to a four-necked round flask, respectively. Adding NaOH to reaction system and adjust the pH to 6-7, then SDS (1.0 g, 3.47 mmol) was added into reaction flask. The hydrophobic monomer FEA (0.35 g) was added and mixture was stirred under N_2 atmosphere until a clear homogeneous mixture was observed. The initiator potassium persulfate (0.1 g, 3.47 mmol) was added and the total monomer concentration in mixture was kept at 20wt%. The polymerization was conducted at 55°C for 4 h to obtain a gel polymer. The polymer were washed with acetone and extracted with ethanol for several times. The product was dried at 60°C under vacuum for 24 h.

^{1}H-NMR(600 MHz, D_2O) δ (ppm): 3.19-3.68 (d, $-CH_2-SO_3H$ and e), 2.31 (c, $-C-CH_3$), 2.20 (b, -CH-), 1.49-1.74 (a、e、f, $-CH_2-$); ^{13}C-NMR (150 MHz, D_2O) δ (ppm): 179.92 (a, C=O), 58.84 (d, C-O), 52.26 (b, C-S), 39.49 (c, C-N), 34.83 (e, $-CH_2-$), 25.85 (f,-CH-), 16.18 (g, $-CH_3$); ^{19}F-NMR (564 MHz, D_2O) δ (ppm): 79.33 ($-CF_3$), 122.25 ($-CF_2$).

Synthesis of HPAAS

AM (9.43 g, 130.0 mmol), AMPS (6.87 g, 33.0 mmol), deionized water (64.40 g) were added to a four-necked round flask, respectively. Adding NaOH to reaction system and adjust the pH to 6-7, then SDS (0.98 g, 3.40 mmol) was added into reaction flask. The hydrophobic monomer ST (0.07 g) was added and mixture was stirred under N_2 atmosphere until a clear homogeneous mixture was observed. The initiator potassium persulfate (0.098 g, 0.36 mmol) was added and the total monomer concentration in mixture was kept at 20wt%. The polymerization was conducted at 55°C for 4 h to obtain a gel polymer. The polymer were washed with acetone and extracted with ethanol for several times. The product was dried at 60°C under vacuum for 24 h.

^{1}H-NMR(600 MHz, D_2O) δ (ppm): 7.74 (e、f、g, -C_6H_5), 3.17-3.61 (d, -CH_2-SO_3H), 2.30 (c, -C-CH_3), 2.19 (b, -CH-), 1.4-1.79 (a, -CH_2-); ^{13}C-NMR (150 MHz, D_2O) δ (ppm): 180.18 (a, C=O),127.85 (h, -C_6H_5), 53.81 (b, C-S), 41.93 (c, C-N), 34.98 (e, -CH_2-), 26.50 (f,-CH-), 14.69 (g, -CH_3).

Synthesis of HPAAD

AM (9.43 g, 130.0 mmol), AMPS (6.87 g, 33.0 mmol), deionized water (65.15 g) were added to a four-necked round flask, respectively. Adding NaOH to reaction system and adjust the pH to 6-7, then SDS (0.99 g, 3.43 mmol) was added into reaction flask. The hydrophobic monomer dodecyl acrylate (0.26 g) was added and mixture was stirred under N_2 atmosphere until a clear homogeneous mixture was observed. The initiator potassium persulfate (0.10 g, 0.37 mmol) was added and the total monomer concentration in mixture was kept at 20wt%. The polymerization was conducted at 55°C for 4 h to obtain a gel polymer. The polymer were washed with acetone and extracted with ethanol for several times. The product was dried at 60°C under vacuum for 24 h.

^{1}H-NMR(600 MHz, D_2O) δ (ppm): 3.19-3.64 (d, -CH_2-SO_3H and e), 2.31 (c, -C-CH_3), 2.19 (b, -CH-), 1.46-1.74 (a、e、f, -CH_2-), 1.46 (f, -CH_3); ^{13}C-NMR (150 MHz, D_2O) δ (ppm): 179.02 (a, C=O), 56.05 (d, C-O), 51.46 (b, C-S), 42.00 (c, C-N), 33.44 (e, -CH_2-), 23.07 (f,-CH-), 15.00 (g, -CH_3).
